# Editorial Note

**DOI:** 10.1155/1995/42425

**Published:** 1995


					HPB Surgery, 1995, Vol. 8, p.
Reprints available directly from the publisher
Photocopying permitted by license only
(C) 1995 Harwood Academic Publishers GmbH
Printed in Malaysia
Editorial Note
There are two additional features in this issue of HPB Surgery from now on the authors of papers reviewed in
HPB International are invited to reply to the review, and Professor Bismuth's comments on the reviews of his
paper will appear in the next issue.
Secondly in this issue we are publishing a list of international meetings which will be of interest to the HPB
community. If organisers ofconferences would like their meetings mentioned in this section please write to me at
the HPB Editorial Office.
I also want to stimulate a lively correspondence section on any matters pertaining to the subject and papers
published in the journal; letters should be no longer than 300 words. All authors must sign the letter and the
authors of the original paper will be invited to reply to the correspondence before it is published.
MCAP

				

